# (3′*R*)-3′-Benzyl-2′,3′-dihydro-1*H*-spiro­[indole-3,1′-naphtho­[2,3-*c*]pyrrole]-2,4′,9′-trione

**DOI:** 10.1107/S1600536812036227

**Published:** 2012-08-25

**Authors:** Garima Sharma, S. Vasanth Kumar, Habibah A. Wahab, Mohd Mustaqim Rosli, Hoong-Kun Fun

**Affiliations:** a Department of Chemistry, Karunya University, Coimbatore, India; b School of Pharmaceutical Sciences, Universiti Sains Malaysia, 11800 USM, Penang, Malaysia; c Malaysian Institute of Pharmaceuticals and Nutraceuticals, Ministry of Science, Technology and Innovation, Halaman Bukit Gambir, 11700 Bayan Lepas, Penang, Malaysia; d X-ray Crystallography Unit, School of Physics, Universiti Sains Malaysia, 11800 USM, Penang, Malaysia

## Abstract

In the title compound, C_26_H_18_N_2_O_3_, the maximum deviations from planarity for the tetra­hydro-1*H*-naphtho­[2,3-*c*]pyrrole and indoline rings systems are 0.091 (1) and 0.012 (2) Å, respectively. These ring systems make a dihedral angle of 89.95 (6)° with each other and they make dihedral angles of 73.42 (8) and 71.28 (9)°, respectively, with the benzene ring. In the crystal, inversion dimers linked by pairs of N—H⋯O hydrogen bonds generate *R*_2_^2^(8) loops and C—H⋯O inter­actions connect the dimers into corrugated sheets lying parallel to the *bc* plane.

## Related literature

For a related structure, see: Sharma *et al.* (2012 ▶). For the biological activity of naphtho­quinones, see: Babula *et al.* (2007 ▶). For 1,3-cyclo­addition reactions involving naphtho­quinones, see: Chen *et al.* (2011 ▶). For the stability of the temperature controller used in the data collection, see: Cosier & Glazer (1986 ▶).
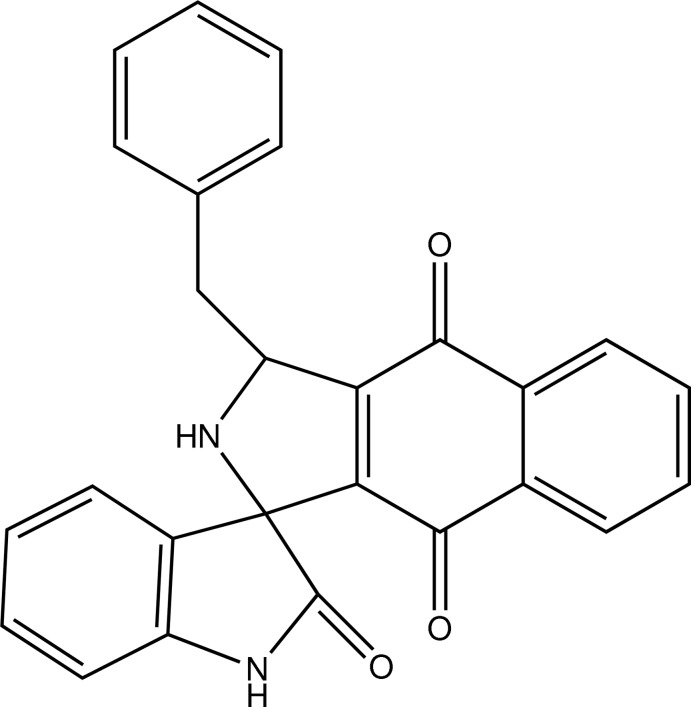


## Experimental

### 

#### Crystal data

C_26_H_18_N_2_O_3_
*M* *_r_* = 406.42
Monoclinic, 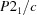

*a* = 10.2317 (4) Å
*b* = 26.2823 (8) Å
*c* = 7.8406 (3) Å
β = 109.122 (2)°
*V* = 1992.10 (12) Å^3^
*Z* = 4
Mo *K*α radiation
μ = 0.09 mm^−1^
*T* = 100 K
0.36 × 0.20 × 0.10 mm

#### Data collection

Bruker SMART APEXII CCD diffractometer
Absorption correction: multi-scan (*SADABS*; Bruker, 2009 ▶) *T*_min_ = 0.968, *T*_max_ = 0.992
19577 measured reflections
5742 independent reflections
3338 reflections with *I* > 2σ(*I*)
*R*_int_ = 0.069

#### Refinement

*R*[*F* ^2^ > 2σ(*F* ^2^)] = 0.064
*wR*(*F* ^2^) = 0.178
*S* = 1.02
5742 reflections
288 parameters
H atoms treated by a mixture of independent and constrained refinement
Δρ_max_ = 0.45 e Å^−3^
Δρ_min_ = −0.27 e Å^−3^

### 

Data collection: *APEX2* (Bruker, 2009 ▶); cell refinement: *SAINT* (Bruker, 2009 ▶); data reduction: *SAINT*; program(s) used to solve structure: *SHELXTL* (Sheldrick, 2008 ▶); program(s) used to refine structure: *SHELXTL*; molecular graphics: *SHELXTL*; software used to prepare material for publication: *SHELXTL* and *PLATON* (Spek, 2009 ▶).

## Supplementary Material

Crystal structure: contains datablock(s) I, global. DOI: 10.1107/S1600536812036227/hb6939sup1.cif

Structure factors: contains datablock(s) I. DOI: 10.1107/S1600536812036227/hb6939Isup2.hkl

Supplementary material file. DOI: 10.1107/S1600536812036227/hb6939Isup3.cml

Additional supplementary materials:  crystallographic information; 3D view; checkCIF report

## Figures and Tables

**Table 1:** Hydrogen-bond geometry (Å, °)

*D*—H⋯*A*	*D*—H	H⋯*A*	*D*⋯*A*	*D*—H⋯*A*
N2—H1*N*2⋯O3^i^	0.95 (3)	1.92 (3)	2.840 (2)	164 (2)
C5—H5*A*⋯O1^ii^	0.95	2.41	3.038 (3)	123

Symmetry codes: (i) 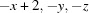; (ii) 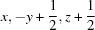.

**Fig. 1.**
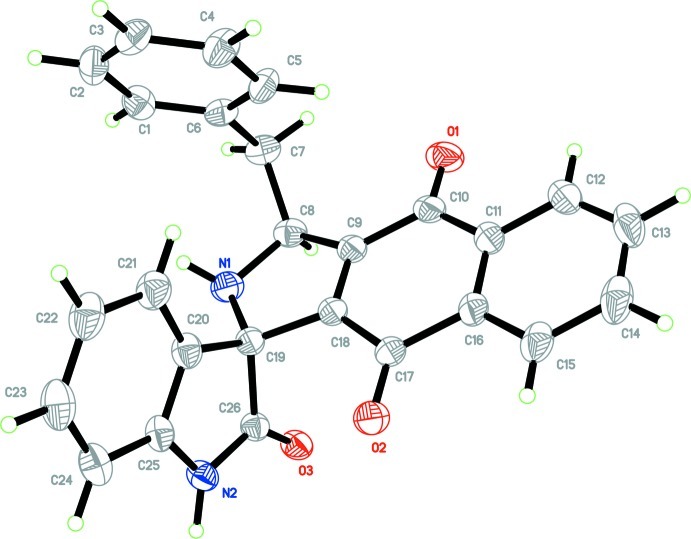
The molecular structure of (I), showing 50% probability displacement ellipsoids.

**Fig. 2.**
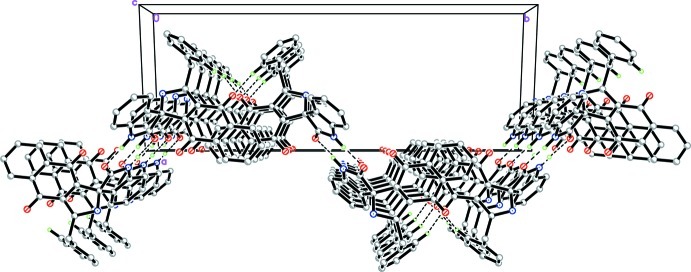
The crystal packing of (I). Dashed lines indicate hydrogen bonds. H atoms not involved in the hydrogen bond interactions have been omitted for clarity.

## supplementary crystallographic information

### Comment

This is a continuation of our recently published work (Sharma *et al.*, 2012). Naphthoquinones are known to possess various biological properties (Babula *et al.*, 2007). Recently, there also have been a few efforts to conduct 1, 3-cycloaddition involving naphthoquinones (Chen *et al.*, 2011).

In the title compound, Fig. 1, all parameters are within normal ranges and comparable with the previously reported structure (Sharma *et al.*, 2012). The tetrahydro-1*H*-naphtho[2,3-*c*]pyrrole (N1/C8—C19) and indoline (N2/C19—C26) rings are close to planar with the maximum deviations of 0.091 (2) Å for atom C8 and 0.012 (2) Å for atom C26. The two rings make a dihedral angle of 89.95 (6)° with each other and these two rings make dihedral angles of 73.42 (8)° and 71.28 (9)° with the benzene ring(C1—C6), respectively.

In the crystal, N2—H1N2···O3^i^ and C5—H5A···O1^ii^ (Table 1) connect the molecules into corrugated sheets parallel to the *bc*-plane (Fig. 2).

### Experimental

A mixture of isatin (0.147 g, 1 mmol), *L*-phenylalanine (0.165 g, 1 mmol) and 1,4-naphthoquinone (0.158 g, 1 mmol) was refluxed in methanol (6 ml) until the starting material was completely utilized (monitored by thin layer chromatography). After leaving the resultant concoction to stand for 1 h, the reaction solid was washed with cool water (3 × 2.5 ml) and cool ethanol (3 × 0.5 ml). The crude reaction solid was re-crystallized from hot methanol to afford the pure product (80% yield) as yellow plates.

### Refinement

N bound H atom were located from a difference Fourier map and freely refined. The remaining H atoms were positioned geometrically and refined using a riding model with C—H = 0.95–1.00 Å and *U*_iso_(H) = 1.2*U*_eq_(C).

### Crystal data

**Table:**

C_26_H_18_N_2_O_3_	*F*(000) = 848
*M**_r_* = 406.42	*D*_x_ = 1.355 Mg m^−^^3^
Monoclinic, *P*2_1_/*c*	Mo *K*α radiation, λ = 0.71073 Å
Hall symbol: -P 2ybc	Cell parameters from 2917 reflections
*a* = 10.2317 (4) Å	θ = 3.6–30.0°
*b* = 26.2823 (8) Å	µ = 0.09 mm^−^^1^
*c* = 7.8406 (3) Å	*T* = 100 K
β = 109.122 (2)°	Plate, yellow
*V* = 1992.10 (12) Å^3^	0.36 × 0.20 × 0.10 mm
*Z* = 4	

### Data collection

**Table:**

Bruker SMART APEXII CCD diffractometer	5742 independent reflections
Radiation source: fine-focus sealed tube	3338 reflections with *I* > 2σ(*I*)
Graphite monochromator	*R*_int_ = 0.069
φ and ω scans	θ_max_ = 30.0°, θ_min_ = 3.1°
Absorption correction: multi-scan (*SADABS*; Bruker, 2009)	*h* = −14→14
*T*_min_ = 0.968, *T*_max_ = 0.992	*k* = −36→36
19577 measured reflections	*l* = −10→10

### Refinement

**Table:**

Refinement on *F*^2^	Primary atom site location: structure-invariant direct methods
Least-squares matrix: full	Secondary atom site location: difference Fourier map
*R*[*F*^2^ > 2σ(*F*^2^)] = 0.064	Hydrogen site location: inferred from neighbouring sites
*wR*(*F*^2^) = 0.178	H atoms treated by a mixture of independent and constrained refinement
*S* = 1.02	*w* = 1/[σ^2^(*F*_o_^2^) + (0.0823*P*)^2^ + 0.2374*P*] where *P* = (*F*_o_^2^ + 2*F*_c_^2^)/3
5742 reflections	(Δ/σ)_max_ < 0.001
288 parameters	Δρ_max_ = 0.45 e Å^−^^3^
0 restraints	Δρ_min_ = −0.27 e Å^−^^3^

### Special details

**Table:**

Experimental. The crystal was placed in the cold stream of an Oxford Cryosystems Cobra open-flow nitrogen cryostat (Cosier & Glazer, 1986) operating at 100.0 (1) K.
Geometry. All e.s.d.'s (except the e.s.d. in the dihedral angle between two l.s. planes) are estimated using the full covariance matrix. The cell e.s.d.'s are taken into account individually in the estimation of e.s.d.'s in distances, angles and torsion angles; correlations between e.s.d.'s in cell parameters are only used when they are defined by crystal symmetry. An approximate (isotropic) treatment of cell e.s.d.'s is used for estimating e.s.d.'s involving l.s. planes.
Refinement. Refinement of *F*^2^ against ALL reflections. The weighted *R*-factor *wR* and goodness of fit *S* are based on *F*^2^, conventional *R*-factors *R* are based on *F*, with *F* set to zero for negative *F*^2^. The threshold expression of *F*^2^ > σ(*F*^2^) is used only for calculating *R*-factors(gt) *etc*. and is not relevant to the choice of reflections for refinement. *R*-factors based on *F*^2^ are statistically about twice as large as those based on *F*, and *R*- factors based on ALL data will be even larger.

### Fractional atomic coordinates and isotropic or equivalent isotropic displacement parameters (Å^2^)

**Table:**

	*x*	*y*	*z*	*U*_iso_*/*U*_eq_	
O1	0.63086 (17)	0.24596 (6)	−0.1639 (2)	0.0425 (4)	
O2	0.99431 (16)	0.12903 (5)	0.32957 (19)	0.0322 (3)	
O3	0.90923 (16)	0.05795 (5)	−0.09244 (19)	0.0303 (3)	
N1	0.6184 (2)	0.08098 (7)	−0.0952 (2)	0.0323 (4)	
N2	0.89813 (19)	0.01158 (6)	0.1530 (2)	0.0279 (4)	
C1	0.2987 (2)	0.10602 (8)	−0.0342 (3)	0.0326 (5)	
H1A	0.2716	0.0806	−0.1253	0.039*	
C2	0.2518 (2)	0.10324 (8)	0.1128 (3)	0.0350 (5)	
H2A	0.1916	0.0765	0.1204	0.042*	
C3	0.2923 (2)	0.13924 (8)	0.2481 (3)	0.0334 (5)	
H3A	0.2610	0.1372	0.3494	0.040*	
C4	0.3785 (2)	0.17823 (8)	0.2351 (3)	0.0358 (5)	
H4A	0.4074	0.2030	0.3283	0.043*	
C5	0.4237 (2)	0.18162 (8)	0.0863 (3)	0.0322 (5)	
H5A	0.4818	0.2090	0.0778	0.039*	
C6	0.3846 (2)	0.14535 (7)	−0.0502 (3)	0.0277 (4)	
C7	0.4355 (2)	0.14789 (8)	−0.2108 (3)	0.0326 (5)	
H7A	0.4226	0.1830	−0.2588	0.039*	
H7B	0.3772	0.1252	−0.3065	0.039*	
C8	0.5876 (2)	0.13280 (8)	−0.1709 (3)	0.0292 (5)	
H8A	0.6077	0.1336	−0.2874	0.035*	
C9	0.6946 (2)	0.16499 (7)	−0.0368 (3)	0.0259 (4)	
C10	0.7108 (2)	0.22060 (8)	−0.0448 (3)	0.0301 (5)	
C11	0.8288 (2)	0.24382 (7)	0.0988 (3)	0.0299 (5)	
C12	0.8434 (3)	0.29669 (8)	0.1069 (3)	0.0393 (6)	
H12A	0.7767	0.3175	0.0231	0.047*	
C13	0.9546 (3)	0.31879 (9)	0.2366 (4)	0.0457 (7)	
H13A	0.9632	0.3548	0.2431	0.055*	
C14	1.0541 (3)	0.28861 (9)	0.3576 (3)	0.0432 (6)	
H14A	1.1317	0.3040	0.4442	0.052*	
C15	1.0407 (2)	0.23625 (8)	0.3524 (3)	0.0351 (5)	
H15A	1.1087	0.2158	0.4358	0.042*	
C16	0.9273 (2)	0.21352 (7)	0.2246 (3)	0.0284 (4)	
C17	0.9102 (2)	0.15717 (7)	0.2248 (3)	0.0250 (4)	
C18	0.7843 (2)	0.13671 (7)	0.0901 (3)	0.0243 (4)	
C19	0.7453 (2)	0.08120 (7)	0.0663 (3)	0.0247 (4)	
C20	0.7310 (2)	0.05476 (7)	0.2298 (3)	0.0286 (4)	
C21	0.6447 (2)	0.06580 (8)	0.3289 (3)	0.0351 (5)	
H21A	0.5821	0.0936	0.2975	0.042*	
C22	0.6529 (3)	0.03456 (9)	0.4769 (3)	0.0410 (6)	
H22A	0.5956	0.0412	0.5484	0.049*	
C23	0.7448 (3)	−0.00613 (9)	0.5190 (3)	0.0410 (6)	
H23A	0.7485	−0.0270	0.6194	0.049*	
C24	0.8312 (3)	−0.01737 (8)	0.4200 (3)	0.0348 (5)	
H24A	0.8933	−0.0454	0.4501	0.042*	
C25	0.8228 (2)	0.01413 (7)	0.2749 (3)	0.0276 (4)	
C26	0.8609 (2)	0.04958 (7)	0.0286 (3)	0.0248 (4)	
H1N1	0.551 (3)	0.0684 (11)	−0.059 (4)	0.065 (9)*	
H1N2	0.968 (3)	−0.0123 (9)	0.156 (3)	0.045 (7)*	

### Atomic displacement parameters (Å^2^)

**Table:**

	*U*^11^	*U*^22^	*U*^33^	*U*^12^	*U*^13^	*U*^23^
O1	0.0417 (10)	0.0389 (9)	0.0539 (10)	0.0140 (7)	0.0252 (8)	0.0197 (7)
O2	0.0299 (9)	0.0331 (8)	0.0342 (8)	0.0021 (6)	0.0110 (7)	0.0021 (6)
O3	0.0354 (9)	0.0290 (7)	0.0322 (8)	0.0062 (6)	0.0188 (7)	0.0033 (5)
N1	0.0287 (10)	0.0337 (10)	0.0345 (10)	0.0016 (8)	0.0103 (8)	−0.0007 (7)
N2	0.0326 (10)	0.0244 (9)	0.0290 (9)	0.0045 (7)	0.0135 (8)	0.0022 (6)
C1	0.0332 (13)	0.0290 (11)	0.0310 (11)	−0.0018 (9)	0.0043 (9)	−0.0041 (8)
C2	0.0327 (13)	0.0322 (11)	0.0382 (12)	−0.0071 (9)	0.0092 (10)	0.0050 (9)
C3	0.0292 (12)	0.0418 (12)	0.0321 (11)	−0.0005 (9)	0.0141 (9)	0.0014 (9)
C4	0.0338 (13)	0.0426 (12)	0.0345 (12)	−0.0083 (10)	0.0161 (10)	−0.0112 (9)
C5	0.0269 (12)	0.0357 (11)	0.0384 (12)	−0.0089 (9)	0.0164 (10)	−0.0084 (9)
C6	0.0214 (10)	0.0328 (10)	0.0275 (10)	0.0036 (8)	0.0058 (8)	−0.0008 (8)
C7	0.0288 (12)	0.0405 (12)	0.0285 (11)	0.0042 (9)	0.0092 (9)	−0.0013 (8)
C8	0.0302 (12)	0.0353 (11)	0.0253 (10)	0.0051 (9)	0.0133 (9)	0.0022 (8)
C9	0.0282 (11)	0.0264 (10)	0.0292 (10)	0.0035 (8)	0.0176 (8)	0.0033 (7)
C10	0.0316 (12)	0.0301 (10)	0.0373 (11)	0.0073 (9)	0.0233 (10)	0.0089 (8)
C11	0.0363 (12)	0.0254 (10)	0.0397 (12)	0.0003 (8)	0.0287 (10)	0.0015 (8)
C12	0.0479 (15)	0.0277 (11)	0.0605 (15)	0.0024 (10)	0.0424 (13)	0.0036 (9)
C13	0.0623 (18)	0.0263 (11)	0.0695 (17)	−0.0098 (11)	0.0503 (15)	−0.0101 (11)
C14	0.0493 (15)	0.0423 (13)	0.0521 (15)	−0.0191 (11)	0.0358 (13)	−0.0163 (11)
C15	0.0357 (13)	0.0368 (12)	0.0420 (12)	−0.0088 (9)	0.0253 (10)	−0.0075 (9)
C16	0.0314 (11)	0.0270 (10)	0.0362 (11)	−0.0033 (8)	0.0240 (10)	−0.0035 (8)
C17	0.0260 (11)	0.0268 (10)	0.0283 (10)	0.0011 (8)	0.0171 (8)	−0.0003 (7)
C18	0.0261 (11)	0.0253 (9)	0.0268 (10)	0.0016 (8)	0.0161 (8)	0.0001 (7)
C19	0.0268 (11)	0.0241 (9)	0.0266 (10)	0.0010 (8)	0.0132 (8)	−0.0002 (7)
C20	0.0344 (12)	0.0281 (10)	0.0266 (10)	−0.0029 (8)	0.0143 (9)	−0.0004 (8)
C21	0.0396 (14)	0.0348 (11)	0.0369 (12)	−0.0029 (10)	0.0207 (10)	−0.0021 (9)
C22	0.0489 (16)	0.0439 (13)	0.0389 (13)	−0.0104 (11)	0.0262 (11)	−0.0029 (10)
C23	0.0563 (17)	0.0388 (12)	0.0316 (12)	−0.0112 (11)	0.0193 (11)	0.0026 (9)
C24	0.0453 (14)	0.0282 (10)	0.0295 (11)	−0.0045 (9)	0.0103 (10)	0.0025 (8)
C25	0.0346 (12)	0.0234 (9)	0.0270 (10)	−0.0045 (8)	0.0132 (9)	−0.0020 (7)
C26	0.0266 (11)	0.0235 (9)	0.0254 (10)	0.0007 (8)	0.0101 (8)	−0.0015 (7)

### Geometric parameters (Å, º)

**Table:**

O1—C10	1.219 (2)	C9—C10	1.475 (3)
O2—C17	1.225 (2)	C10—C11	1.485 (3)
O3—C26	1.225 (2)	C11—C12	1.397 (3)
N1—C8	1.478 (3)	C11—C16	1.404 (3)
N1—C19	1.487 (3)	C12—C13	1.381 (4)
N1—H1N1	0.89 (3)	C12—H12A	0.9500
N2—C26	1.361 (2)	C13—C14	1.390 (4)
N2—C25	1.413 (3)	C13—H13A	0.9500
N2—H1N2	0.95 (3)	C14—C15	1.382 (3)
C1—C2	1.388 (3)	C14—H14A	0.9500
C1—C6	1.389 (3)	C15—C16	1.395 (3)
C1—H1A	0.9500	C15—H15A	0.9500
C2—C3	1.380 (3)	C16—C17	1.491 (3)
C2—H2A	0.9500	C17—C18	1.474 (3)
C3—C4	1.378 (3)	C18—C19	1.508 (3)
C3—H3A	0.9500	C19—C20	1.507 (3)
C4—C5	1.391 (3)	C19—C26	1.552 (3)
C4—H4A	0.9500	C20—C21	1.384 (3)
C5—C6	1.391 (3)	C20—C25	1.389 (3)
C5—H5A	0.9500	C21—C22	1.402 (3)
C6—C7	1.513 (3)	C21—H21A	0.9500
C7—C8	1.536 (3)	C22—C23	1.390 (4)
C7—H7A	0.9900	C22—H22A	0.9500
C7—H7B	0.9900	C23—C24	1.387 (3)
C8—C9	1.505 (3)	C23—H23A	0.9500
C8—H8A	1.0000	C24—C25	1.386 (3)
C9—C18	1.336 (3)	C24—H24A	0.9500
			
C8—N1—C19	110.48 (16)	C11—C12—H12A	119.9
C8—N1—H1N1	112.6 (19)	C12—C13—C14	120.3 (2)
C19—N1—H1N1	106 (2)	C12—C13—H13A	119.8
C26—N2—C25	111.33 (17)	C14—C13—H13A	119.8
C26—N2—H1N2	122.5 (15)	C15—C14—C13	120.3 (2)
C25—N2—H1N2	126.2 (15)	C15—C14—H14A	119.8
C2—C1—C6	121.01 (19)	C13—C14—H14A	119.8
C2—C1—H1A	119.5	C14—C15—C16	119.9 (2)
C6—C1—H1A	119.5	C14—C15—H15A	120.0
C3—C2—C1	120.3 (2)	C16—C15—H15A	120.0
C3—C2—H2A	119.9	C15—C16—C11	119.91 (19)
C1—C2—H2A	119.9	C15—C16—C17	119.7 (2)
C4—C3—C2	119.5 (2)	C11—C16—C17	120.43 (19)
C4—C3—H3A	120.3	O2—C17—C18	121.16 (17)
C2—C3—H3A	120.3	O2—C17—C16	122.93 (19)
C3—C4—C5	120.4 (2)	C18—C17—C16	115.90 (17)
C3—C4—H4A	119.8	C9—C18—C17	123.79 (18)
C5—C4—H4A	119.8	C9—C18—C19	110.90 (18)
C6—C5—C4	120.7 (2)	C17—C18—C19	125.13 (17)
C6—C5—H5A	119.6	N1—C19—C20	114.94 (17)
C4—C5—H5A	119.6	N1—C19—C18	103.35 (15)
C1—C6—C5	118.11 (19)	C20—C19—C18	115.91 (15)
C1—C6—C7	120.54 (18)	N1—C19—C26	110.06 (15)
C5—C6—C7	121.34 (19)	C20—C19—C26	101.98 (15)
C6—C7—C8	114.88 (17)	C18—C19—C26	110.74 (16)
C6—C7—H7A	108.5	C21—C20—C25	121.35 (19)
C8—C7—H7A	108.5	C21—C20—C19	129.63 (19)
C6—C7—H7B	108.5	C25—C20—C19	109.01 (17)
C8—C7—H7B	108.5	C20—C21—C22	117.8 (2)
H7A—C7—H7B	107.5	C20—C21—H21A	121.1
N1—C8—C9	103.11 (16)	C22—C21—H21A	121.1
N1—C8—C7	112.82 (18)	C23—C22—C21	119.9 (2)
C9—C8—C7	116.98 (17)	C23—C22—H22A	120.0
N1—C8—H8A	107.8	C21—C22—H22A	120.0
C9—C8—H8A	107.8	C24—C23—C22	122.4 (2)
C7—C8—H8A	107.8	C24—C23—H23A	118.8
C18—C9—C10	121.58 (19)	C22—C23—H23A	118.8
C18—C9—C8	111.86 (17)	C25—C24—C23	116.9 (2)
C10—C9—C8	126.40 (18)	C25—C24—H24A	121.6
O1—C10—C9	121.2 (2)	C23—C24—H24A	121.6
O1—C10—C11	122.08 (19)	C24—C25—C20	121.6 (2)
C9—C10—C11	116.72 (18)	C24—C25—N2	128.7 (2)
C12—C11—C16	119.4 (2)	C20—C25—N2	109.71 (17)
C12—C11—C10	119.4 (2)	O3—C26—N2	126.73 (19)
C16—C11—C10	121.17 (18)	O3—C26—C19	125.32 (17)
C13—C12—C11	120.1 (2)	N2—C26—C19	107.95 (16)
C13—C12—H12A	119.9		
			
C6—C1—C2—C3	−1.3 (3)	C10—C9—C18—C19	−179.17 (16)
C1—C2—C3—C4	0.7 (3)	C8—C9—C18—C19	−3.6 (2)
C2—C3—C4—C5	0.5 (3)	O2—C17—C18—C9	−174.50 (18)
C3—C4—C5—C6	−1.1 (3)	C16—C17—C18—C9	5.6 (3)
C2—C1—C6—C5	0.7 (3)	O2—C17—C18—C19	0.2 (3)
C2—C1—C6—C7	179.80 (19)	C16—C17—C18—C19	−179.66 (16)
C4—C5—C6—C1	0.5 (3)	C8—N1—C19—C20	130.56 (17)
C4—C5—C6—C7	−178.6 (2)	C8—N1—C19—C18	3.3 (2)
C1—C6—C7—C8	−105.2 (2)	C8—N1—C19—C26	−115.02 (17)
C5—C6—C7—C8	73.9 (3)	C9—C18—C19—N1	0.2 (2)
C19—N1—C8—C9	−5.1 (2)	C17—C18—C19—N1	−175.10 (17)
C19—N1—C8—C7	−132.25 (18)	C9—C18—C19—C20	−126.48 (19)
C6—C7—C8—N1	57.3 (2)	C17—C18—C19—C20	58.2 (2)
C6—C7—C8—C9	−62.1 (2)	C9—C18—C19—C26	118.02 (18)
N1—C8—C9—C18	5.4 (2)	C17—C18—C19—C26	−57.3 (2)
C7—C8—C9—C18	129.81 (19)	N1—C19—C20—C21	−61.8 (3)
N1—C8—C9—C10	−179.28 (18)	C18—C19—C20—C21	58.8 (3)
C7—C8—C9—C10	−54.8 (3)	C26—C19—C20—C21	179.2 (2)
C18—C9—C10—O1	177.88 (19)	N1—C19—C20—C25	118.20 (19)
C8—C9—C10—O1	3.0 (3)	C18—C19—C20—C25	−121.20 (19)
C18—C9—C10—C11	−2.1 (3)	C26—C19—C20—C25	−0.8 (2)
C8—C9—C10—C11	−177.06 (17)	C25—C20—C21—C22	0.0 (3)
O1—C10—C11—C12	5.0 (3)	C19—C20—C21—C22	−180.0 (2)
C9—C10—C11—C12	−174.99 (17)	C20—C21—C22—C23	−0.5 (3)
O1—C10—C11—C16	−173.87 (18)	C21—C22—C23—C24	0.4 (4)
C9—C10—C11—C16	6.1 (3)	C22—C23—C24—C25	0.2 (3)
C16—C11—C12—C13	0.7 (3)	C23—C24—C25—C20	−0.6 (3)
C10—C11—C12—C13	−178.23 (18)	C23—C24—C25—N2	179.8 (2)
C11—C12—C13—C14	1.2 (3)	C21—C20—C25—C24	0.5 (3)
C12—C13—C14—C15	−1.7 (3)	C19—C20—C25—C24	−179.47 (18)
C13—C14—C15—C16	0.4 (3)	C21—C20—C25—N2	−179.80 (19)
C14—C15—C16—C11	1.5 (3)	C19—C20—C25—N2	0.2 (2)
C14—C15—C16—C17	−177.37 (18)	C26—N2—C25—C24	−179.7 (2)
C12—C11—C16—C15	−2.0 (3)	C26—N2—C25—C20	0.7 (2)
C10—C11—C16—C15	176.84 (18)	C25—N2—C26—O3	178.67 (19)
C12—C11—C16—C17	176.85 (17)	C25—N2—C26—C19	−1.2 (2)
C10—C11—C16—C17	−4.3 (3)	N1—C19—C26—O3	58.9 (3)
C15—C16—C17—O2	−2.4 (3)	C20—C19—C26—O3	−178.64 (19)
C11—C16—C17—O2	178.71 (18)	C18—C19—C26—O3	−54.7 (3)
C15—C16—C17—C18	177.45 (17)	N1—C19—C26—N2	−121.22 (17)
C11—C16—C17—C18	−1.4 (2)	C20—C19—C26—N2	1.2 (2)
C10—C9—C18—C17	−3.8 (3)	C18—C19—C26—N2	125.13 (17)
C8—C9—C18—C17	171.80 (17)		

### Hydrogen-bond geometry (Å, º)

**Table:**

*D*—H···*A*	*D*—H	H···*A*	*D*···*A*	*D*—H···*A*
N2—H1*N*2···O3^i^	0.95 (3)	1.92 (3)	2.840 (2)	164 (2)
C5—H5*A*···O1^ii^	0.95	2.41	3.038 (3)	123

Symmetry codes: (i) −*x*+2, −*y*, −*z*; (ii) *x*, −*y*+1/2, *z*+1/2.
